# Editorial: Noncaloric artificial sweeteners and their impact on human health

**DOI:** 10.3389/fnut.2024.1461624

**Published:** 2024-08-19

**Authors:** Galileo Escobedo, Mariana Buranelo Egea, Ernesto Roldan-Valadez, Christopher Peter-Corpe, Nallely Bueno-Hernández

**Affiliations:** ^1^Laboratory of Immunometabolism, Research Division, General Hospital of Mexico “Dr. Eduardo Liceaga”, Mexico City, Mexico; ^2^Biocomopunds and Nutrition Laboratory, Goiano Federal Institute (IF GOIANO), Rio Verde, Brazil; ^3^Division of Research, Instituto Nacional de Rehabilitacion “Luis Guillermo Ibarra Ibarra”, Mexico City, Mexico; ^4^Department of Radiology, I.M. Sechenov First Moscow State Medical University (Sechenov University), Moscow, Russia; ^5^Department of Nutritional Sciences, King's College London, London, United Kingdom; ^6^Proteomics and Metabolomics Laboratory, Research Division, General Hospital of Mexico “Dr. Eduardo Liceaga”, Mexico City, Mexico

**Keywords:** non-caloric sweeteners, microbiome, diabetes, glucose intolerance, sweet taste receptor, dietary additives, artificial sweeteners

## Introduction

Non-caloric artificial sweeteners (NAS), including saccharin, sucralose, aspartame, acesulfame-K, advantame, and neotame, are widely used as dietary supplements worldwide (1). In recent years, NAS consumption has increased among people of all ages and socioeconomic backgrounds, particularly in countries with Western diets, where reducing body mass index (BMI) has become aesthetically imperative beyond health considerations. The food industry leverages NAS to create low-calorie foods and beverages with minimal glycemic impact and low cost, while maintaining the palatability of sucrose and other caloric sweeteners (2–4).

The body cannot metabolize most NAS, leading to incomplete absorption and various changes in the gastrointestinal tract, including the gut microbiome and carbohydrate metabolism (5). However, recent literature shows contradictory results regarding the potential harms or benefits of NAS (3, 6, 7). Notably, there is significant concern about the toxicity and interaction of non-metabolized compounds (metabolites) with microbiota, as shown in preclinical models and human studies. Research teams around the globe have focused mainly acute metabolism and endocrine responses to NAS, but the evidence remains limited and inconsistent (2–4, 8); moreover, recently the World Health Organization (WHO) suggested that NAS not be used as a means of achieving weight control or reducing the risk of non-communicable diseases (conditional recommendation) (9). For this reason, we edited a Research Topic within Frontiers in Nutrition to explore the impact of NAS on human health.

**Figure 1:**
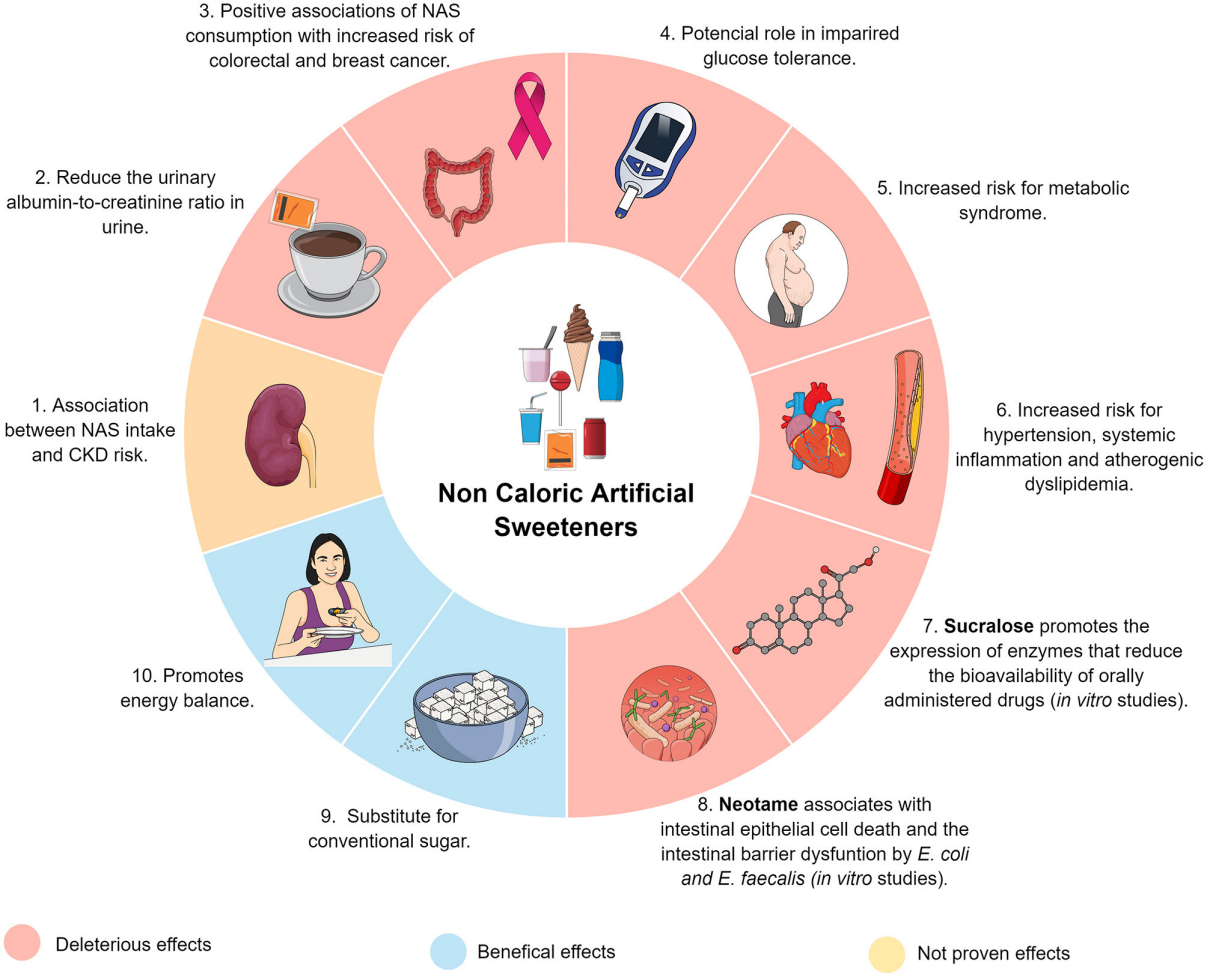
Graphical representation of the effects of NAS reported in published studies. In red the harmful effects, in blue the positive effects and in yellow the unproven effects.

We received numerous high-quality submissions from renowned research groups worldwide, including clinical trials, and experimental studies in humans, cell models, and animals. As depicted in Figure 1, we included three original articles and two review manuscripts, which we will discuss further.

### Findings from the included studies

Ran et al. contributed to the Research Topic by presenting original data on the effect of NAS on kidney function and the development of chronic kidney disease (CKD) development. Using data from the National Health and Nutrition Examination Survey (NHANES) from 2003 to 2006, they conducted an analysis of covariance and weighted adjusted logistic regression to examine the association between NAS consumption and the likelihood of developing CKD, along with its impact on kidney function. Additionally, in this work, the authors employed Mendelian randomization methods and the primary analysis methods included instrumental variable analysis with inverse variance weighting and robust adjusted profile score. The study included 20,470 participants, of whom 1,257 were ultimately analyzed. The adjusted logistic regression models showed no significant association between NAS consumption and CKD risk. The summary odds ratios (OR) for each unit change in genetically predicted CKD risk were not statistically significant. However, the addition of NAS to coffee was associated with a reduction in the urinary albumin-to-creatinine ratio. In conclusion, the study shows no association between NAS intake and CKD risk. Nevertheless, these results need to be further validated by larger samples and Mendelian randomization analyses (Singh S. et al.). This study emphasizes that although NAS have no direct effect on kidney function, there is evidence suggesting a link to certain types of cancer, particularly in the presence of obesity. Obesity, as measured by BMI, emerges as an important mediating factor, suggesting that a person's weight status can significantly influence the impact of NAS consumption on health. Therefore, further research is essential to fully understand these effects and the potential interactions between NAS consumption, body weight, and cancer risk.

## In-depth analysis of additional studies

Although Ran et al.'s study indicated NAS do not directly affect kidney function, there is evidence suggesting a link to certain types of cancer, especially in the presence of obesity. BMI emerges as an important mediating factor, indicating that a person's weight status can significantly influence the impact of NAS consumption. Thus, further research is essential to fully understand these effects and the potential interactions between NAS consumption, body weight, and cancer risk. In this regard, Jin et al. showed in the Research Topic a work that explored the genetic causality between NAS consumption and the risk of obesity-related cancers (ORCs), using Mendelian randomization to reduce external confounders. The authors conducted a series of analyses to assess the causal associations between NAS consumption and ORC risk using data from the genome-wide association study (GWAS). In this study, the authors conducted a broad MR analysis, which is a comprehensive and detailed investigation of the association between NAS consumption and the risk of ORCs. They found a positive association of NAS consumption with an increased risk of colorectal cancer (CRC) and breast cancer. However, in multivariable analyses, the authors identified BMI as a key mediator in the association between NAS consumption and CRC, as obesity is a pronounced risk factor for various cancers, especially those of obesity-related origin such as esophageal, pancreatic, and colorectal cancers (Jin et al.).

On the other hand, Shil et al. used a model combining the Caco-2 intestinal epithelium cell line and microbiota species such as *Escherichia coli* and *Enterococcus faecalis* to explore novel pathogenic effects of neotame on intestinal cell function, microbiome metabolism, and gut pathogenicity. Neotame is the strongest of the sweeteners. It is a derivative of a dipeptide compound (aspartic acid and phenylalanine) and was developed as a sweetener with a high degree of sweetness to add to a variety of foods. The authors investigated interactions between intestinal epithelium cells and microbiota, demonstrating that NAS can considerably accumulate in the intestine. Given the concentrations of sweeteners in commercially available products, it is plausible that exposing the gut to up to 2 mM sweeteners, as occurs after consuming a diet soft drink, may damage the intestinal epithelium, especially with neotame at concentrations of 0.1–50 mM. Moreover, the neotame caused epithelial cell death and intestinal barrier disruption at concentrations as low as 0.1 mM and 1 μM, respectively. Co-culture studies with *E. coli* or *E. faecalis* revealed pathogenic effects of neotame at 100 μM, a concentration much lower than that found in food and drinks, and even the acceptable daily intake limits (Angelin et al.). This study highlights the effects of neotame on intestinal cell function and microbiome, sparking new discussion about appropriate NAS concentrations in commercially available foods and beverages.

Singh S. et al. challenged the conventional wisdom suggesting that sucralose contributes to weight control by examining evidence from *in vivo* studies on gut microbiota and metabolic syndrome. Their review article outlines the impact of sucralose on carbohydrate metabolism and obesity development. The authors argue that while NAS are a low-calorie alternative, their impact on sweet taste receptor expression and glucose cellular metabolism raises concerns about their potential role in promoting weight gain and obesity. In addition, they highlight the numerous negative effects associated with the consumption of sucralose, such as an increased risk of developing metabolic syndrome, liver inflammation, and cardiovascular disease. The report also addresses the adverse effects of sucralose on the bioavailability of orally ingested medications. It points out that sucralose can trigger the release of chlorinated aromatic polycyclic hydrocarbons (CI-CAPH) in the body, which are compounds potentially associated with a carcinogenic effect. This information raises concerns about the wider health effects of sucralose consumption (Singh S. et al.).

Finally, Angelin et al. presented a comprehensive review manuscript on NAS's effects on diabetes. This review suggests that NAS may benefit diabetes care, particularly in weight loss and blood glucose control, by influencing energy balance when used as alternatives to conventional sugars. Nevertheless, the authors also describe that NAS can contribute to hypertension, systemic inflammation, impaired glucose tolerance, atherogenic dyslipidemia, kidney disease, and prothrombotic state. They emphasize that further research is essential to develop targeted interventions that mitigate the risks associated with NAS consumption (Singh S. et al.).

## Conclusion

This Research Topic demonstrates the multiple actions of NAS consumption on human health, ranging from inducing obesity, diabetes, and CKD to intestinal dysfunction and various types of cancer. These findings underscore the need for continued in-depth investigations to fully understand the health implications of NAS consumption.
